# Response-adaptive breast cancer care in the grey zones: integrating ER and HER2 targeted PET, FDG PET/CT under immunotherapy, and ctDNA kinetics

**DOI:** 10.37349/etat.2026.1002398

**Published:** 2026-08-16

**Authors:** Jovana Zivanovic, Jules Zhang-Yin

**Affiliations:** Fondazione Policlinico Universitario Agostino Gemelli IRCCS, Italy; ^1^Centre for Nuclear Medicine and PET, University Clinical Centre of Serbia, 11000 Belgrade, Serbia; ^2^Department of Nuclear Medicine, Clinique Sud Luxembourg, Vivalia, 6700 Arlon, Belgium; ^3^Department of Nuclear Medicine, Centre National PET, Centre Hospitalier de Luxembourg, L-1210 Luxembourg, Luxembourg

**Keywords:** breast cancer, PET/CT, FDG, FES, HER2 imaging, trastuzumab, ctDNA, response-adaptive therapy

## Abstract

Breast cancer management increasingly hinges on decisions made in “grey zones” where tissue sampling is scarce, and tumour biology evolves under therapeutic pressure. This narrative review synthesises evidence on how whole-body imaging biomarkers and circulating tumour DNA (ctDNA) can support response-adaptive pathways at the interface of surgical and systemic care. Four clinically actionable domains are discussed. (a) 16α-[18F]fluoro-17β-estradiol ([18F]FES) positron emission tomography/computed tomography (PET/CT) enables non-invasive, whole-body mapping of functional oestrogen receptor (ER) expression to address receptor discordance, heterogeneous metastases, and selection of endocrine-based strategies. (b) Human epidermal growth factor receptor 2 (HER2)-targeted PET (notably ^89^Zr-trastuzumab, with emerging alternatives) provides whole-body receptor assessment to uncover actionable HER2-positive disease despite HER2-negative primaries, informing anti-HER2 treatment selection when repeat biopsy is infeasible. (c) In triple-negative breast cancer treated with immune checkpoint inhibitors, 18F-fluorodeoxyglucose (18F-FDG) PET/CT offers quantitative response and whole-body burden assessment but requires immunotherapy-aware interpretation (e.g., confirmation strategies for apparent early progression) to mitigate pseudoprogression and dissociated responses, while simultaneously visualising immune-related adverse events. (d) Pairing early metabolic change on FDG PET/CT with ctDNA kinetics is presented as a biologically complementary approach for response monitoring and risk stratification, with potential to inform trial-embedded response-adaptive hypotheses, with ctDNA offering a rapid systemic trajectory and PET providing lesion-level localisation in heterogeneous or oligoprogressive disease. Overall, these tools add value only when linked to prespecified clinical questions and consensus actions; prospective studies are needed to validate standardised, outcome-improving response-adaptive algorithms that integrate imaging and liquid biopsy. Key implementation challenges include tracer availability, harmonised acquisition/reconstruction, threshold definition, and avoiding overtesting. Near-term impact may be greatest in problem-solving and in trial-embedded decision rules that operationalise biomarker-guided care.

## Introduction

The management of breast cancer is not a straightforward process. A plethora of pivotal choices are to be made at various points along the pathway, including the extent to which neoadjuvant therapy should be pursued prior to surgery, the potential benefits and risks of broadening locoregional treatment in cases of uncertain metastatic risk, and the management of lesions that demonstrate variable responses or appear to alter biological characteristics over time. These ‘grey zones’ are indicative of genuine tumour evolution, encompassing phenomena such as clonal selection, receptor conversion and microenvironmental influences. Additionally, they highlight the practical limitations of repeated biopsy procedures. In that context, whole-body, repeatable biomarkers are attractive because they can sample disease beyond a single lesion and can be revisited when the clinical question changes. Positron emission tomography (PET) coupled with computed tomography (CT) provides functional information regarding metabolic activity or target engagement, with the additional advantage of spatial resolution. In contrast, circulating tumour DNA (ctDNA) offers a blood-based, systemic trajectory that may anticipate radiological change and can hint at emerging resistance. However, it should be noted that the presence of additional information does not inherently guarantee optimal decision-making. It is imperative that each test is meticulously designed to address a specific and well-defined clinical query and subsequently linked to a consensus action. This approach is pivotal in averting the pitfalls of unnecessary expenditure, complexity, and the potential for both false reassurance and false alarm. The following sections present four focused contributions: oestrogen receptor (ER) functional imaging, human epidermal growth factor receptor 2 (HER2)-targeted PET, FDG PET/CT under immunotherapy in triple-negative breast cancer (TNBC) and combined metabolic/molecular monitoring. These contributions demonstrate the potential applications of these tools and identify the areas where further research is necessary to achieve response-adaptive, clinically significant care.

The level of evidence varies considerably across the topics reviewed. Some applications are supported by meta-analyses or larger clinical series, whereas others rely on small feasibility cohorts, retrospective exploratory studies, or extrapolation from other tumour types. This distinction is clinically important because diagnostic or prognostic association does not necessarily establish clinical utility; biomarker-guided treatment change requires prospective evidence showing improved outcomes, reduced toxicity, or better resource use.

For clarity, the revised discussion ranks each biomarker domain by evidentiary maturity. FES PET/CT has the most mature evidence base among the targeted PET approaches discussed here, supported by meta-analytic data and increasing clinical experience in selected ER-positive scenarios. FDG PET/CT has established oncologic utility and a substantial response-assessment literature, but immunotherapy-specific interpretation in TNBC remains less validated. HER2-targeted PET is biologically compelling and clinically promising, but much of the evidence remains derived from small, single-centre or feasibility studies. Combined FDG PET/CT and ctDNA kinetics are best regarded as a conceptual and trial-ready strategy, because prospective outcome-improving algorithms have not yet been established.

To improve readability, heterogeneity, biopsy limitations, and response-adaptive decision-making are treated here as shared cross-cutting themes rather than being restated in full within each modality-specific section. Subsequent sections therefore focus on how each biomarker addresses a specific clinical question, while acknowledging that none should be used in isolation or outside a clearly defined decision pathway.

## Review methodology

This manuscript is a narrative review rather than a systematic review or meta-analysis. Relevant references were identified through targeted searches of PubMed/MEDLINE, Scopus, Web of Science, Google Scholar and citation tracking of key articles, focusing on publications up to early 2026 that addressed FES PET/CT, HER2-targeted PET, FDG PET/CT in immunotherapy-treated breast cancer, and ctDNA monitoring. Search terms combined breast cancer with modality-specific terms such as FES PET, ER PET, HER2 PET, trastuzumab PET, pertuzumab PET, FDG PET immunotherapy, TNBC, ctDNA, liquid biopsy, response monitoring, escalation, and de-escalation. Priority was given to meta-analyses, systematic reviews, prospective clinical studies, guideline-relevant evidence and studies with direct management implications; smaller feasibility studies and retrospective cohorts were included when they addressed emerging tracers, rare clinical scenarios, or conceptual response-adaptive frameworks. Because the review is narrative, the included evidence should be interpreted as a critical synthesis rather than an exhaustive systematic search, and potential selection bias remains a limitation.

The level of evidence varies considerably across the topics reviewed. Some applications are supported by meta-analyses or larger clinical series, whereas others rely on small feasibility cohorts, retrospective exploratory studies, or extrapolation from other tumour types. This distinction is clinically important because diagnostic or prognostic association does not necessarily establish clinical utility; biomarker-guided treatment change requires prospective evidence showing improved outcomes, reduced toxicity, or better resource use.

## 16α-[18F]fluoro-17β-estradiol ([18F]FES) PET/CT for whole-body ER mapping in ER-positive disease

The management of breast cancer is increasingly characterised by “grey zones” that sit at the interface between surgery and systemic therapy. These include when to intensify or de-escalate treatment, how to interpret spatial and temporal tumour heterogeneity, and how to individualise decisions when tissue sampling is incomplete or misleading. In ER-positive disease, these controversies are amplified because ER is both the key predictive biomarker for endocrine strategies and a moving target under therapeutic pressure, clonal evolution, and metastatic spread. Within this special issue focused on contentious topics and paradigm shifts, ER imaging with [18F]FES PET/CT stands out as a pragmatic, non-invasive method to map functional ER expression across the whole body, addressing decision bottlenecks that otherwise force clinicians to rely on limited biopsies, imperfect surrogates, or historical receptor status.

Throughout this review, we distinguish higher-level evidence (systematic reviews/meta-analyses, prospective trials, and guideline/consensus statements) from feasibility or small observational cohorts. Where evidence is exploratory, we explicitly frame conclusions as hypothesis-generating and highlight key limitations, controversies and standardisation gaps. The evidence base for [18F]FES PET/CT has matured alongside its clinical use: meta-analytic data link ER-targeted uptake to endocrine responsiveness [1], and comparative analyses show that [18F]FES complements rather than replaces glucose metabolism imaging with 18F-fluorodeoxyglucose (18F-FDG) [2]. The literature now speaks directly to real-world dilemmas that shape modern pathways, including receptor conversion and discordance, oligometastatic management, and treatment selection amid heterogeneity [3]. Radiology- and oncology-facing reviews emphasise that [18F]FES PET is not simply another staging scan; it is a biomarker test whose value depends on pre-test probability, the specific question asked (e.g., “is there sufficient ER functionality anywhere to justify endocrine escalation?”), and awareness of pitfalls such as physiologic hepatic uptake and limited interpretability in liver-dominant disease [4]. This frames a controversy central to this special issue: whether “more imaging” risks overdiagnosis and overtreatment, or whether biomarker imaging enables de-escalation by preventing futile systemic therapy and avoiding unnecessary surgery or biopsies when biology is already unfavourable.

In contemporary practice, ER-targeted PET has been evaluated in staging and problem-solving workflows to identify ER-positive sites that may be occult or equivocal on standard imaging and to support management changes that directly affect the surgical/systemic balance [5]. This is especially relevant in settings where disease extent is difficult to judge and local therapy decisions are contentious, such as invasive lobular carcinoma and low-metabolic phenotypes; [18F]FES can reveal ER-positive burden when FDG is less informative, influencing multidisciplinary choices around breast-conserving strategies, nodal surgery, and systemic escalation [6]. Many reviews argue that [18F]FES PET may become a “decision driver” in metastatic HR+/HER2− disease, where the key issue is not historical ER positivity but whether ER remains sufficiently functional across dominant lesions to justify endocrine-based strategies rather than chemotherapy or other targeted approaches [7]. Umbrella syntheses place [18F]FES within a broader shift toward precision pathways that integrate imaging biomarkers with liquid biopsy and AI-assisted stratification, while also highlighting ongoing challenges in evidence quality, indication selection, and standardised reporting [8].

Biologically, [18F]FES offers whole-body quantification of ER functionality, which is most valuable when heterogeneity makes the “single-lesion truth” unreliable; prospective and open-access datasets continue to explore lesion-level uptake patterns and outcomes [9]. Implementation debates now also include resource use and downstream consequences: health-economic modelling suggests [18F]FES PET/CT after failed biopsy or inconclusive immunohistochemistry, or when biopsy is infeasible, may reduce repeat biopsies and inappropriate therapies, with potential cost savings and improved quality-adjusted outcomes [10]. Pragmatic regional reviews reinforce that although availability is variable, the clinical niche is becoming clearer: problem-solving ER status, mapping heterogeneity, and guiding endocrine decisions when conventional tools are equivocal [11]. A particularly actionable dimension is identification of heterogeneity linked to endocrine resistance, such as [18F]FES-negative sites within an otherwise ER-positive phenotype that predict poorer outcomes under CDK4/6 inhibitor plus endocrine therapy, supporting earlier consideration of alternative systemic strategies [12]. Beyond baseline stratification, [18F]FES has served as a pharmacodynamic biomarker in trials tracking biological effects of combination approaches and linking them to response signals [13].

Complementarity with FDG is illustrated in metastatic lobular disease, where [18F]FES delineates ER-avid burden while FDG highlights more aggressive components, supporting nuanced treatment selection in settings where “one size fits all” staging is controversial [14]. In earlier-phase contexts, [18F]FES PET/CT can influence staging and management in ways that intersect with escalation/de-escalation debates: detecting unsuspected metastatic disease may avert non-beneficial surgery, while demonstrating widespread ER-positive burden may support endocrine-first strategies that could reduce surgical morbidity [15]. Mechanistic confidence is supported by evidence of [18F]FES binding specificity for ER-positive lesions [16], and the ability to quantify ER blockade/suppression in vivo adds a dimension to debates about sequencing and duration of endocrine agents or SERDs, as [18F]FES can reflect target engagement and help explain discordant clinical courses [17]. Earlier clinical observations in acquired hormone-resistant metastatic disease further reinforce why “historic ER positivity” is often insufficient for today’s heterogeneity-driven decision-making [18]. Collectively, [18F]FES PET challenges over-reliance on single-site biopsy, reveals heterogeneity that drives controversy in choosing endocrine therapy versus chemotherapy, and provides an evidence-based tool to individualise care; while leaving active debates around standardisation, equity of access, and embedding into adaptive pathways (including AI-supported decision tools) without amplifying overtesting. Finally, [18F]FES also illustrates a broader point: pathology is essential but sparse, a limitation that becomes even more consequential when the therapeutic target is binary, as with HER2, setting the stage for HER2-directed PET to map actionable expression when re-biopsy is impractical or unsafe (Supplementary Figure S1).

The [18F]FES PET/CT literature includes systematic reviews and meta-analyses supporting associations between ER-targeted uptake and endocrine responsiveness, alongside expanding prospective and real-world series. However, many studies remain single-centre and heterogeneous in patient selection, timing relative to endocrine agents, and reporting thresholds. Key controversies include limited interpretability in liver-dominant disease due to physiologic hepatic uptake, variable access across regions, and uncertainty regarding how best to operationalise lesion-level heterogeneity into standardised, outcome-improving response-adaptive treatment algorithms.

## HER2-targeted PET imaging to detect actionable receptor heterogeneity

HER2-targeted PET imaging, most prominently with ^89^Zr-trastuzumab PET/CT, has emerged as a whole-body approach to interrogate HER2 biology in metastatic breast cancer, particularly in clinical “grey zones” where tissue sampling is incomplete, discordant, or impractical. The central rationale is straightforward: HER2 is a treatment-defining biomarker, and single-site assessment may not reflect whole-body disease when receptor expression is heterogeneous or changes over time. In this context, HER2-targeted PET aims to detect clinically relevant HER2-positive disease that might be missed by conventional sampling, including HER2-positive metastases in patients whose primary tumour was classified as HER2-negative [19]. Because HER2-directed therapy can substantially alter treatment strategy and prognosis in appropriate patients, a non-invasive method to map HER2 expression across all lesions is conceptually attractive [20].

The clinical literature supporting HER2-targeted PET in breast cancer is still dominated by feasibility studies and small observational cohorts, but these reports collectively provide a plausible signal that whole-body imaging can reveal actionable heterogeneity. Early work with ^89^Zr-trastuzumab demonstrated that a subset of patients with HER2-negative primary disease showed positive tracer uptake at metastatic sites; in some cases, subsequent management incorporated HER2-targeted therapy, illustrating the potential of imaging to uncover a treatable phenotype not captured by the original pathology [19]. Follow-up cohorts remained small but reinforced the same theme: occasional biopsy-confirmed HER2-positive metastases can exist despite a HER2-negative primary classification, supporting the biological plausibility of receptor conversion and sampling error [21]. In a practical problem-solving setting, ^89^Zr-trastuzumab PET/CT has also been reported in patients with equivocal HER2 status after standard work-up; in that context, treatment strategy changes were described, including both initiation of anti-HER2 therapy when uptake supported HER2-positive disease and withholding of planned anti-HER2 therapy when whole-body uptake was not supportive [22]. These observations are important because they move beyond pure feasibility toward potential decision impact; however, the underlying evidence remains exploratory, and the same studies do not establish that imaging-guided decisions improve outcomes compared with a biopsy-led approach.

A key reason HER2-targeted PET remains compelling is that receptor instability is not theoretical. Tissue-based studies have shown that clinically used biomarkers [including ER, progesterone receptor (PR), and HER2] may change during tumour progression when comparing primary tumours with metastases [23]. Discordance has also been reported between primary tumours and axillary lymph nodes, and conversion from HER2-negative to HER2-positive status may open additional therapeutic options, potentially affecting quality of life and survival in some cases [24]. Imaging approaches therefore align with a well-recognised biological problem: the “truth” of receptor status is not always static, and a single biopsy may capture only part of the disease. In this setting, a whole-body method could theoretically complement pathology by identifying the most informative lesions to sample or by highlighting heterogeneity that might influence systemic strategy.

Nevertheless, interpreting HER2-targeted PET requires caution because PET signal does not map perfectly onto immunohistochemistry. In an imaging study evaluating ^89^Zr-trastuzumab PET/CT, higher uptake was generally observed in HER2-positive compared with HER2-negative lesions (with recognised limitations for liver metastases), and a standardized uptake value (SUV)max threshold was proposed with good positive predictive value but only moderate sensitivity [24]. This illustrates both promise and constraint: uptake may help identify HER2-positive disease, but it is not a perfect discriminator, and any single numerical threshold is not yet sufficiently standardised across centres, protocols, scanners, and patient populations. Further, lack of correlation between radiotracer uptake and HER2 expression intensity on immunohistochemistry has been described, emphasising that uptake reflects more than receptor density alone (e.g., delivery/perfusion, antibody kinetics, prior therapies) [19]. Qualitative interpretation may sometimes perform comparably to established modalities used for response evaluation in broader breast cancer contexts, but feasibility results cannot substitute for prospective validation in defined clinical decision pathways [25]. In other words, HER2-targeted PET can demonstrate biologic plausibility and may aid problem-solving, but the field is still working toward reproducible thresholds and validated clinical action rules.

Beyond ^89^Zr-trastuzumab, several alternative HER2-targeted tracers have been investigated, each with potential niche advantages but similarly early evidence. ^64^Cu-DOTA-trastuzumab has been discussed mainly in relation to predicting benefit from trastuzumab emtansine (T-DM1); practical drawbacks include less convenient whole-body coverage, potential overlap with adjacent blood vessels, and the implications of a shorter half-life for delayed imaging [26]. At the same time, early uptake at 1–2 days after injection has been suggested as a way to identify patients unlikely to benefit from T-DM1, positioning imaging as a potential biomarker of delivery/target engagement rather than simply a static receptor map [26]. Another putative advantage is brain metastasis assessment, where tissue sampling is notoriously difficult; small series have reported visualisation of brain metastases with ^64^Cu-DOTA-trastuzumab, raising the hypothesis that trastuzumab-based tracers may access lesions behind a disrupted blood-brain barrier [27]. Other tracers include ^89^Zr-pertuzumab, conceptually similar in aiming to map HER2 expression and explored for monitoring therapy response (including after T-DM1 exposure) [28, 29], and ^68^Ga-ABY-025, which has been reported to discriminate HER2-positive from HER2-negative lesions and to capture whole-body HER2 expression in heterogeneous disease [30]. Collectively, these studies illustrate an expanding tracer landscape but do not yet provide a cohesive, standardised framework for routine clinical use.

Practical considerations substantially constrain near-term implementation. First, availability remains limited: these tracers are typically produced and used in specialised centres, and widespread access is unlikely in the short term. Second, workflow and radiation exposure are non-trivial, especially for ^89^Zr-labelled antibodies that require delayed imaging and may deliver a higher radiation dose than conventional PET tracers, making repeated examinations less attractive in routine practice. Third, interpretive pitfalls remain relevant, including physiological and heterogeneous background tracer uptake, which may complicate lesion assessment [31]. Finally, trastuzumab itself is rarely used as monotherapy; it is typically combined with other agents, complicating attempts to attribute clinical benefit to a single target engagement signal and making it harder to translate imaging into a clear, validated action without a prespecified algorithm [24].

For these reasons, the most defensible near-term clinical positioning is to treat HER2-targeted PET as a problem-solving or trial-embedded tool. In carefully selected cases—such as suspected receptor conversion, discordant pathology across sites, inability to safely re-biopsy, or the need to prioritise the best lesion for confirmatory tissue sampling—whole-body HER2 imaging may provide incremental information that supports multidisciplinary discussion [19, 21, 22, 24]. However, what remains uncertain is the key question of clinical utility: how often PET changes management appropriately, and whether PET-guided treatment selection improves patient outcomes compared with standard tissue-based assessment and guideline-driven care. Until prospective interventional trials demonstrate outcome benefit using prespecified PET-guided decision rules, treatment-changing observations from existing cohorts should be framed as exploratory and hypothesis-generating rather than practice-shaping. In summary, HER2-targeted PET provides a biologically compelling window into whole-body receptor heterogeneity, but its clinical readiness is limited by small cohort evidence, lack of harmonised thresholds, logistical constraints, and absence of outcome-driven validation; therefore it should be used selectively, with explicit acknowledgement of uncertainty, rather than as a routine stand-alone determinant of HER2-directed treatment adaptation [19–31].

## FDG PET/CT for immunotherapy assessment in TNBC

Immune checkpoint inhibitors (ICIs) have reshaped the therapeutic landscape of TNBC, first in metastatic disease and increasingly in high-risk early TNBC (eTNBC) when combined with chemotherapy. Their clinical impact has sharpened a long-standing imaging controversy: how to judge benefit when tumour size is an unreliable surrogate of response. Under cytotoxics and many targeted agents, early assessment still leans on anatomic shrinkage (RECIST-based paradigms), whereas ICIs introduce immune kinetics and immune-related adverse events (irAEs) that can produce atypical patterns: pseudoprogression, dissociated response, hyperprogression, and durable responses complicating decisions to stop or continue therapy if conventional imaging is used in isolation. In this setting, 18F-FDG PET/CT is attractive because it provides quantitative whole-body readouts of tumour glycolytic activity and burden while also depicting irAEs as metabolically active inflammatory lesions. The issue in TNBC is therefore not detectability, but whether FDG PET/CT can deliver robust, standardised, and actionable information for immunotherapy decision-making: supporting escalation or de-escalation without prompting premature discontinuation of effective therapy or unnecessary continuation of futile regimens. A narrative synthesis on PET/CT in breast cancer treated with immunotherapy argues that FDG PET/CT is well positioned because it couples quantitative lesion assessment with whole-body mapping of both cancer and inflammation, while acknowledging that breast cancer evidence remains less mature than in melanoma or lung cancer and requires careful criteria selection and prospective validation [32].

Atypical response patterns sit at the centre of immunotherapy imaging. Pseudoprogression describes early apparent worsening; transient lesion enlargement or new lesions followed by regression, plausibly driven by immune cell infiltration, oedema, and delayed kinetics; dissociated response reflects heterogeneous lesion-level sensitivity, with some sites regressing while others progress; hyperprogression represents accelerated growth soon after ICI initiation with rapid clinical decline; and durable responses may persist after discontinuation [32]. In metastatic TNBC, where ICIs are frequently paired with chemotherapy, misclassifying early “progression” can trigger a damaging regimen change, making immunotherapy-aware criteria essential. Several PET-adapted frameworks have been proposed, including immune PERCIST (iPERCIST), PERCIMT, imPERCIST5, and PECRIT, aiming to detect true progression early while protecting against premature cessation in pseudoprogression [32]. iPERCIST is particularly pragmatic: if PERCIST-defined progressive metabolic disease is seen at the first on-treatment scan, it is labelled unconfirmed progressive metabolic disease (uPMD), and a repeat scan after 4–8 weeks is recommended to confirmed progressive metabolic disease (cPMD) in clinically stable patients [32]. The same review notes that some apparent early progressions later behave as pseudoprogression or dissociated response, and that continuing ICI, sometimes with local treatment of oligoprogressive lesions, may benefit selected patients [32], reinforcing that FDG PET/CT functions best as decision support anchored to an immunotherapy-aware interpretative framework.

However, caution is required when applying these frameworks to TNBC. Many immunotherapy-adapted imaging criteria were developed largely from experience in melanoma, lung cancer, and other tumour types, and breast cancer-specific validation remains limited. Therefore, in TNBC, these criteria should be considered interpretative aids rather than validated treatment-decision algorithms. Apparent early metabolic progression should be interpreted in conjunction with symptoms, laboratory findings, treatment timing, and conventional imaging, and confirmation imaging may be appropriate in clinically stable patients before changing systemic therapy.

TNBC is a compelling arena for functional imaging because of its aggressive behaviour, marked heterogeneity, and typically high baseline FDG avidity, which improves lesion detectability and quantification. A systematic review (up to February 2024) concluded that PET imaging is “essential” for initial staging in TNBC (localised and metastatic), can influence management through upstaging in a subset, and can help predict pathological complete response (pCR) during neoadjuvant chemotherapy through serial changes in FDG uptake (often SUVmax), while also highlighting expanding roles for volumetric metrics [metabolic tumour volume/total lesion glycolysis (MTV/TLG)] and radiomics for prognostication and response prediction [33]. Quantitative FDG metrics are commonly grouped into single-lesion intensity [SUVmax, SUV normalized to lean body mass (SUL)peak], whole-body tumour burden (MTV, TLG, lesion counts), and heterogeneity features (textural/radiomic indices capturing intra- and inter-lesion variability). Baseline intensity and burden correlate with prognosis across TNBC cohorts: in metastatic TNBC, MTV correlates with overall survival [34]; in early-stage TNBC, baseline PET features have been linked to disease-free survival and can refine prognostic groups when integrated with biological markers (e.g., EGFR) [35]; and analyses focusing on MTV, TLG, and entropy have proposed PET-based identification of higher-risk patients in whom escalation could be considered [36]. These baseline observations provide a rationale for exploring whether early on-treatment metabolic changes and heterogeneity dynamics could refine decisions about continuing ICIs, adding local therapy for oligoprogression, or switching strategy.

Heterogeneity is particularly relevant to immunotherapy because both immune response and tumour evolution are intrinsically heterogeneous. In a dedicated metastatic TNBC cohort treated with first-line immunotherapy plus chemotherapy, baseline FDG PET/CT heterogeneity indices were associated with shorter progression-free survival, and an inter-tumour heterogeneity index remained independently predictive in multivariable analysis [37]. This supports the concept that a single “hottest lesion” metric may miss biologic diversity driving mixed responses and early progression under ICIs. Response monitoring, however, remains contentious: while functional response may precede morphologic change and PET can be advantageous in skeletal-dominant disease, PET guidance for monitoring metastatic breast cancer outside immunotherapy is not universally adopted. Methodological work nevertheless supports PERCIST feasibility and interpretability. A retrospective evaluation found PERCIST feasible in most metastatic breast cancer patients and suggested that using a “nadir” scan as reference can help visualise fluctuations and potentially support earlier recognition of non-effective toxic therapy [38]. Another study comparing EORTC criteria and PERCIST reported high agreement and showed metabolic response categories predicting overall survival [39]. These foundations are directly relevant because immunotherapy-modified criteria such as iPERCIST build on PERCIST while adding safeguards (notably confirmation scans) to mitigate misclassification with pseudoprogression and dissociated responses [32] (Supplementary Figure S2).

In eTNBC, where immunotherapy is often delivered in the neoadjuvant setting, pCR remains a key endpoint. FDG PET/CT has long been used to predict early metabolic response and pCR in aggressive subtypes, though thresholds and timepoints vary. A retrospective study in locally advanced TNBC reported baseline and post-neoadjuvant PET predicting pathological response and clinical outcome, with ΔSUVmax cut-offs associated with disease-free survival differences [40]. More broadly, early reductions in SUV metrics after one or two cycles have been observed across subtypes, and modelling suggests early SUV reduction can discriminate pCR, particularly when applied to both the primary tumour and axillary regions [41]. While not always immunotherapy-specific, these studies provide a quantitative scaffold for contemporary regimens combining ICIs with chemotherapy. The tension is practical: early metabolic non-response may argue for escalation or switching, but immunotherapy-aware frameworks caution against labelling early progression without confirmation, and fixed surgical timelines can make delayed confirmation difficult, highlighting the need for standardised PET response criteria and validated decision rules in immunotherapy-treated TNBC.

Radiomics and machine learning may add signal beyond conventional SUV metrics by extracting high-dimensional descriptors of intensity distribution, texture, and shape that may approximate microenvironmental complexity and heterogeneity. A co-clinical approach using subtype-matched patient-derived xenografts identified FDG PET radiomic signatures that outperformed SUV measures for predicting and assessing neoadjuvant response [42]. A prospective cohort in stage I–III breast cancer receiving neoadjuvant chemotherapy reported preliminary evidence that PET radiomic features from the primary lesion predict pCR, including analyses encompassing TNBC [43]. More recent multimodal models combining PET (including radiomics) with histopathologic, genomic, and clinical features have shown proof-of-concept discrimination for pCR prediction in TNBC [44]. These approaches are relevant to immunotherapy because imaging phenotypes may correlate with immune infiltration and microenvironmental features when repeated biopsy is impractical [32], but they also raise familiar controversies around feature stability across scanners and reconstructions, harmonisation, and overfitting, underlining the need for multicentre validation. A further practical advantage of FDG PET/CT under immunotherapy is visualisation of irAEs: inflammatory uptake in organs such as lung, colon, thyroid, pituitary, or joints can explain symptoms and influence continuation decisions [32]. This is particularly relevant when progression is suspected, since distinguishing true progression from inflammatory uptake or identifying irAEs requiring steroids (which may alter tumour metabolism) can materially change interpretation.

Overall, current evidence supports a clinically plausible role for FDG PET/CT in TNBC immunotherapy while clarifying what remains uncertain. Baseline PET can stage and stratify risk (SUV, MTV/TLG, heterogeneity/radiomic metrics), with TNBC-specific data supporting prognostic associations in early and metastatic settings [33–36]. Response assessment under immunotherapy requires criteria aligned with immunotherapy biology; iPERCIST offers an operational approach using unconfirmed/confirmed metabolic progression with short-interval reassessment in stable patients, while alternative criteria exist but require further breast cancer validation [32]. Heterogeneity metrics and radiomics appear promising for capturing diversity underlying mixed responses and early resistance, supported by early metastatic TNBC (mTNBC) data [37] and multiple radiomics/ML studies [42–44]. However, prospective trials that pre-specify PET-derived decision rules (when to continue despite apparent progression, when to add local therapy for dissociated response, when to switch systemic strategy) and demonstrate improved outcomes versus standard pathways remain necessary. Until then, FDG PET/CT can be used as an adjunct for multidisciplinary decision-making when conventional imaging is unclear, when bone-dominant disease complicates RECIST, when clinical deterioration conflicts with apparent stability, or when atypical response patterns are suspected, provided harmonised acquisition/reconstruction and validated response criteria are applied [32, 33]. Immunotherapy can generate non-linear kinetics, and a solitary early scan can mislead; an obvious next step is to add an independent systemic signal. The subsequent combined strategy therefore integrates early metabolic response on FDG PET/CT with ctDNA kinetics to link lesion-level mapping with a molecular trajectory that may evolve ahead of size change and, in selected contexts, provide earlier reassurance or warning than metabolism alone.

Thus, FDG PET/CT under immunotherapy is best viewed as a supportive tool for multidisciplinary interpretation rather than a stand-alone determinant of treatment continuation, escalation, or discontinuation.

TNBC PET evidence is strongest for staging and prognostic associations (including systematic reviews and multiple observational cohorts), whereas PET-guided immunotherapy response adaptation remains less mature. Reported immune-adapted PET frameworks (e.g., iPERCIST and related criteria) are largely extrapolated from other tumour types and require breast cancer-specific prospective validation. Ongoing controversies include optimal timing of early scans during chemo-immunotherapy, risk of misclassification due to pseudoprogression or inflammation, and lack of harmonised thresholds for volumetric/radiomic metrics across scanners and reconstructions.

## Early metabolic response paired with ctDNA for escalation and de-escalation

Early response-adaptive strategies have become a central “controversy meets innovation” theme in breast cancer management: how early should response be measured, which biomarker should arbitrate escalation versus de-escalation, and can repeated imaging be replaced or at least rationalised, without loss of safety? A pragmatic way to frame the debate is to pair biomarkers that interrogate complementary biology: early metabolic response on 18F-FDG PET/CT (tumour viability and whole-body burden) together with ctDNA [molecular burden, clonal dynamics, and minimal residual disease (MRD)]. ctDNA has moved from a conceptual marker to a deployable tool across the breast cancer continuum, with growing evidence for treatment monitoring, prognosis, and relapse prediction [45, 46]. Implementation remains contentious because clinical utility depends on assay strategy (tumour-informed versus tumour-agnostic), sampling cadence and analytical sensitivity, and above all how results are tied to treatment change [45, 47]. In principle, an “ideal” early-response biomarker is rapidly responsive, whole-body and resilient to heterogeneity, quantitative and reproducible, and actionable through pre-specified decisions. ctDNA often satisfies rapid responsiveness and offers a whole-body signal, yet is limited by low tumour fraction, variable shedding and subclonal representation [45, 48]. FDG PET/CT provides lesion-level and organ-level quantification with established reproducibility, but can be confounded by inflammation, partial-volume effects in small lesions, and interpretative difficulties in some patterns such as bone-dominant disease. This is the rationale for combined metabolic-plus-molecular strategies, rather than relying on either modality alone, particularly when the clinical goal is escalation/de-escalation rather than simple prognostication.

It is important to distinguish between biomarkers that are currently clinically actionable and those that remain investigational. In breast cancer, ctDNA has established utility in selected metastatic settings for identifying actionable genomic alterations, whereas its use for longitudinal response monitoring, imaging triage, treatment escalation, or treatment de-escalation remains under prospective evaluation. Similarly, FDG PET/CT provides whole-body functional assessment and may support response evaluation in selected clinical contexts, but PET/ctDNA-guided treatment adaptation has not yet been validated as a standard clinical pathway. Therefore, the combined PET/ctDNA strategy discussed here should be interpreted primarily as a hypothesis-generating and trial-ready framework rather than as an established approach for routine escalation or de-escalation. This distinction is particularly important for de-escalation, where the clinical risk of false reassurance and undertreatment is greater than in escalation strategies.

In practical terms, current validated use cases should be separated from future response-adaptive concepts. Established or near-established applications include using molecular testing to identify actionable alterations in selected metastatic settings and using PET/CT to clarify disease extent or response when the result will answer a defined clinical question. By contrast, changing systemic therapy intensity solely because of early PET/ctDNA kinetics, particularly reducing therapy intensity after apparent biomarker clearance, remains investigational. The review therefore avoids presenting biomarker response as equivalent to a validated treatment decision.

In metastatic breast cancer, professional guidelines already support ctDNA for tumour genotyping to guide targeted therapy, and longitudinal monitoring to anticipate progression before imaging is under active investigation [48]. The plasmaMATCH platform trial illustrates the feasibility and clinical relevance of ctDNA testing to identify actionable alterations and direct mutation-matched therapies in advanced disease, while exposing practical issues such as concordance with tissue, representativeness of archival specimens, and the need for robust assay performance in routine care [49]. The more provocative controversy is whether ctDNA can be used not just to complement imaging, but to triage it. Mouhanna et al. [50] evaluated an ultrasensitive personalised approach in metastatic breast cancer and proposed a ctDNA-guided imaging paradigm in which scans are deferred until a molecular rise is detected; in their early cohort, roughly two-thirds of scans among ctDNA-positive patients could have been avoided without compromising safety, because ctDNA rises typically preceded or coincided with progression. Although not an escalation/de-escalation trial in the strict sense, this provides a concrete proof-of-concept that molecular kinetics can modulate surveillance intensity, a principle that aligns naturally with response-adaptive strategies.

In early-stage disease, the controversy shifts. Clinicians are generally comfortable escalating therapy when there is evidence of insufficient response, while de-escalation based on early biomarkers remains the more contentious step because the downside of undertreatment is relapse. Recent syntheses emphasise that ctDNA’s most potentially transformative role in early breast cancer may be MRD detection and relapse prediction, while noting that heterogeneity of assays and limited interventional evidence constrain routine adoption [51]. Reviews of emerging prospective MRD efforts highlight both opportunity and uncertainty: the ability to detect ctDNA before overt metastasis raises the question of whether (and how) to intervene, but the field still needs proof that ctDNA-guided treatment changes improve outcomes rather than simply stratify prognosis [48]. Across these discussions, a consistent point emerges: ctDNA positivity is prognostic; ctDNA-guided treatment change is still being proven.

FDG PET/CT adds a complementary dimension by providing whole-body functional assessment and quantifying response through changes in SUV metrics, MTV, or TLG. Importantly, imaging-derived tumour burden and blood-based tumour fraction appear to reflect related but non-identical biological dimensions, supporting the logic of pairing them for response-adaptive decisions [52]. Metabolic imaging can reveal discordant responses across sites, a hallmark of heterogeneity and a practical driver of clinical dilemmas (e.g., oligoprogression), while ctDNA may signal molecular non-response even when size change is delayed. Neoadjuvant therapy provides a particularly informative setting for paired monitoring because serial blood sampling is feasible and pathological endpoints such as pCR and residual cancer burden (RCB) provide a reference. Li et al. [53] reported that ctDNA tumour fraction dynamics during neoadjuvant therapy correlate with response categories and with RCB, and that post-neoadjuvant ctDNA relates to recurrence risk in selected comparisons, supporting the idea that molecular clearance is a proxy for deep response and that persistence could trigger escalation. These observations are consistent with broader neoadjuvant ctDNA literature in which early decline or clearance suggests sensitivity, whereas persistence may reflect residual biology that warrants intensification or trial consideration [45, 51]. The increasing use of immunotherapy further sharpens the case for pairing, since inflammatory changes can confound imaging response assessment; liquid biopsy is therefore often discussed as a complementary tool to interpret response and resistance, including under immunotherapy, and to refine selection strategies (still a contested area in breast cancer, particularly outside TNBC) [45].

Conceptually, a response-adaptive framework built on paired biomarkers is straightforward but methodologically demanding. Baseline characterisation includes FDG PET/CT where appropriate and establishment of ctDNA testing. An early on-treatment assessment (often after 1–2 cycles) then evaluates metabolic response (ΔSUV, ΔMTV/TLG) alongside ctDNA kinetics (drop, clearance, persistence, or rise). The critical step is to pre-define actions. If patterns suggest inadequate response, such as limited metabolic reduction and/or persistent or rising ctDNA, escalation is considered, ideally within evidence-based protocols or trials. De-escalation remains the most contentious element because it requires very high negative predictive value. In the near term, the most defensible “de-escalation” may be de-intensified surveillance rather than de-intensified treatment: extending imaging intervals for patients with deep early response (substantial metabolic reduction plus ctDNA clearance), with re-imaging triggered by ctDNA rise, symptoms, or other clinical signals. This mirrors the ctDNA-guided imaging concept explored in metastatic cohorts [50] and directly addresses the practical question of reducing scanning burden without taking on the full risk of treatment de-intensification.

The framework proposed here is conceptual and should be regarded as a structure for future prospective studies rather than a near-term clinical algorithm. For such an approach to become clinically actionable, trials would need to define the imaging protocol, ctDNA assay, sampling timepoints, response thresholds, and treatment actions in advance. They would also need to demonstrate that acting on combined PET/ctDNA results improves clinically meaningful endpoints, such as pathological response, progression-free survival, overall survival, toxicity, quality of life, or healthcare resource use. Until such evidence is available, PET/ctDNA-guided escalation or de-escalation should be restricted to clinical trials or highly selected multidisciplinary decision-making scenarios.

Several controversies must be resolved before such approaches can be standardised. Assay choice (tumour-informed versus broader panels), definitions of “molecular response,” and thresholds for “metabolic non-response” vary widely across studies, limiting transportability and harmonisation [47, 48, 51]. False negatives in ctDNA, low-shedding tumours, sanctuary sites, and small-volume residual disease are particularly problematic when de-escalation is contemplated. Conversely, false positives from clonal haematopoiesis, technical artefacts, or transient fluctuations could drive unnecessary escalation or additional imaging. Ultimately, even when early biomarkers predict outcomes, prospective interventional evidence is required to show that acting on these signals improves survival, quality of life, or resource use; this is the key distinction between prognostic and predictive utility [47, 51]. In summary, pairing early metabolic response with ctDNA offers a rational, whole-body, biologically complementary strategy for navigating the complexity of modern breast cancer care: ctDNA has established roles in genotyping and shows promise for dynamic monitoring in metastatic disease [48–50], with increasing evidence of prognostic relevance in early-stage and neoadjuvant settings [47, 51, 53]. FDG PET/CT contributes spatial resolution and direct measurement of viable tumour burden, particularly useful when heterogeneity or oligoprogression requires localisation. Where cited studies are small or feasibility-based, we use them to illustrate biological plausibility and workflow concepts rather than to imply practice-changing readiness. Prospective interventional trials with prespecified biomarker-guided actions are required before PET/ctDNA-guided treatment adaptation can be considered evidence-based. The current frontier and a central controversy for this special issue is translating these paired signals into actionable, standardised escalation/de-escalation algorithms with proven patient benefit.

The strength of conclusions that can be drawn from combined FDG PET/CT and ctDNA studies remains limited by small cohorts, heterogeneous assays, variable imaging timepoints, and the absence of prospective interventional trials in which treatment is changed according to predefined combined biomarker rules. As a result, combined metabolic and molecular response assessment should currently be viewed as a biologically plausible strategy for risk stratification and trial design, rather than as an evidence-based standard for routine treatment escalation or de-escalation.

## Clinical utility versus biological plausibility

This caution is consistent with the broader emerging-imaging literature in breast cancer. Recent reviews of optical, spectral, and AI-assisted imaging techniques emphasise that promising diagnostic performance must still be translated through standardisation, workflow integration, prospective validation, and demonstration of patient benefit before routine adoption [54, 55]. The same translational standard should be applied to molecular PET and ctDNA-based response-adaptive strategies.

A recurring challenge across these imaging and liquid biopsy strategies is the distinction between biological plausibility, diagnostic performance, prognostic value, and true clinical utility. A biomarker may identify receptor heterogeneity, predict outcome, or detect early molecular change without necessarily improving patient management. Clinical utility requires that the biomarker result leads to a treatment decision that improves outcomes, avoids ineffective therapy, reduces toxicity, or rationalises resource use. This distinction is particularly relevant for HER2-targeted PET, FDG PET/CT under immunotherapy, and PET/ctDNA-guided response adaptation. For HER2-targeted PET, the key unresolved question is not only whether heterogeneous HER2 expression can be detected, but whether PET-directed anti-HER2 treatment selection improves outcomes compared with standard biopsy-guided care. For FDG PET/CT under immunotherapy, the challenge is to avoid both premature discontinuation due to inflammatory or atypical response patterns and inappropriate continuation of ineffective therapy. For PET/ctDNA strategies, the greatest uncertainty concerns de-escalation, where high negative predictive value and prospective evidence are required before treatment intensity can be safely reduced Tables 1, 2, and 3.

**Table 1:** Succinct summary of the response-adaptive breast cancer care in the grey zones.

**Topic**	**Core value**	**Main clinical use case**	**Key limitation**	**Take-home message**
ER-targeted PET ([18F]FES PET/CT)	Whole-body functional ER mapping (captures heterogeneity and receptor discordance)	Suspected ER discordance, mixed endocrine response, biopsy not feasible	Physiologic/background uptake in some organs; not a substitute for histology in all cases Evidence status: supported by meta-analyses and growing clinical experience in selected ER-positive scenarios; not a universal replacement for biopsy.	Best used to answer a specific endocrine decision question (continue/switch endocrine strategy, target biopsy)
HER2-targeted PET	Whole-body HER2 expression assessment beyond a single biopsy	Suspected HER2 conversion/heterogeneity in metastatic disease, especially when tissue is inaccessible	Limited availability, logistics, tracer timing, and standardisation Evidence status: promising but mostly small, feasibility-based or specialised-centre evidence; clinical utility still requires validation.	Useful as a problem-solving biomarker to support anti-HER2 treatment selection or biopsy prioritisation
FDG PET/CT under immunotherapy	Whole-body tumour burden + early metabolic response + potential detection of immune-related adverse events	Response assessment during immune checkpoint inhibitor treatment; equivocal progression	Pseudoprogression/dissociated response can mislead early interpretation Evidence status: Biologically plausible and clinically useful in selected cases, but immunotherapy-specific criteria require TNBC-specific validation.	Interpret with immunotherapy-aware criteria and confirmation strategy
FDG PET/CT + ctDNA kinetics (combined approach)	Combines spatial lesion-level information (PET) with temporal systemic molecular dynamics (ctDNA)	Early escalation/de-escalation decisions, discordant response patterns, oligoprogression	Timing/threshold harmonisation, assay variability, and risk of overtesting Evidence status: conceptual and trial-ready; not yet validated for routine escalation/de-escalation.	Most powerful when embedded in a predefined action algorithm

[18F]FES: 16α-[18F]fluoro-17β-estradiol; ctDNA: circulating tumour DNA; ER: oestrogen receptor; TNBC: triple-negative breast cancer; HER2: human epidermal growth factor receptor 2; PET/CT: positron emission tomography/computed tomography.

**Table 2:** Evidence maturity and clinical readiness of reviewed biomarker applications.

**Biomarker domain**	**More established use**	**Promising but not yet practice-shaping**	**Investigational/conceptual use**
FES PET/CT	Problem-solving whole-body ER mapping in selected ER-positive disease when receptor status or disease extent is uncertain.	Refining endocrine-treatment selection and biopsy targeting in heterogeneous disease.	Routine response-adaptive escalation/de-escalation based on FES patterns alone.
HER2-targeted PET	No broad routine indication; use remains mainly specialised and problem-solving.	Detection of suspected HER2 conversion/heterogeneity when biopsy is infeasible or inconclusive.	PET-directed anti-HER2 treatment selection without prospective outcome validation.
FDG PET/CT under immunotherapy	Whole-body metabolic assessment when conventional imaging is equivocal or bone-dominant disease limits RECIST.	Immunotherapy-aware interpretation of atypical response patterns in TNBC.	Treatment continuation, discontinuation, or escalation based on a single early PET scan.
FDG PET/CT + ctDNA	ctDNA genotyping in selected metastatic settings; PET for spatial disease assessment.	Combined risk stratification and surveillance concepts in trial settings.	Routine treatment de-escalation or escalation based only on early combined biomarker kinetics.

ctDNA: circulating tumour DNA; ER: oestrogen receptor; TNBC: triple-negative breast cancer; HER2: human epidermal growth factor receptor 2; PET/CT: positron emission tomography/computed tomography.

**Table 3:** Practical decision pathway for response-adaptive biomarker use.

**Clinical question**	**Most relevant tool**	**Possible action**	**Caution/evidence status**
Is ER expression still functionally present across disease sites?	FES PET/CT, alongside pathology and standard imaging	Support endocrine-based strategy discussion or identify discordant lesion for biopsy.	Most useful when linked to a specific endocrine decision; not a universal biopsy replacement.
Is HER2 expression heterogeneous or converted when tissue is inaccessible?	HER2-targeted PET where available	Prioritise biopsy site or support multidisciplinary treatment discussion.	Exploratory/problem-solving; outcome benefit of PET-directed therapy remains unproven.
Is apparent early progression during ICI therapy true progression?	FDG PET/CT interpreted with immunotherapy-aware criteria	Correlate clinically; consider confirmation imaging in stable patients before changing therapy.	TNBC-specific validation is limited; inflammatory uptake can mislead.
Is early response sufficient to consider adaptation?	FDG PET/CT plus serial ctDNA in predefined protocols	Escalation or surveillance adaptation only within trials or highly selected MDT contexts.	De-escalation is highest risk and requires prospective outcome validation.

ctDNA: circulating tumour DNA; ER: oestrogen receptor; TNBC: triple-negative breast cancer; HER2: human epidermal growth factor receptor 2; ICI: immune checkpoint inhibitor; PET/CT: positron emission tomography/computed tomography.

## Conclusions

**Figure 1:**
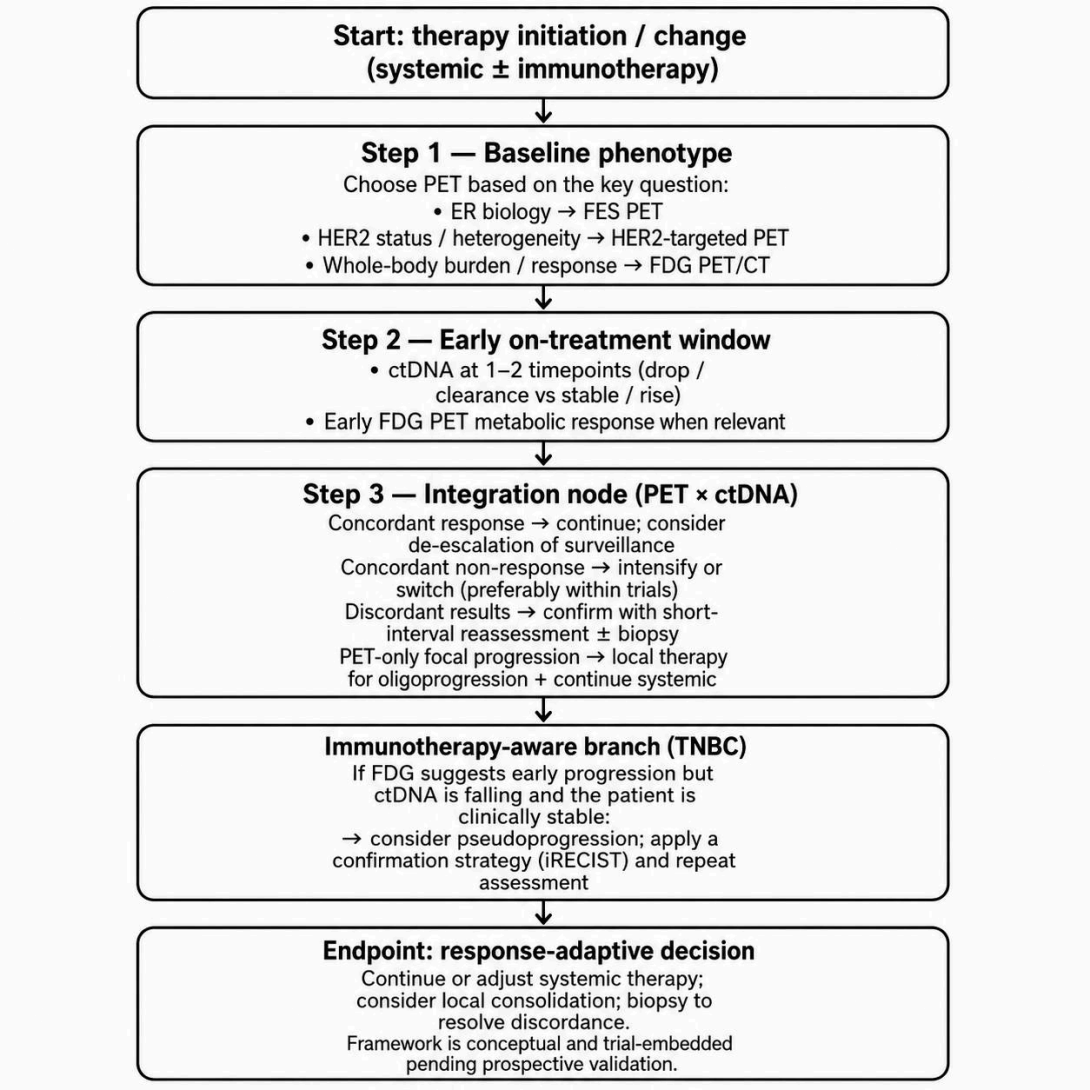
**Integrative schematic approach.** ctDNA: circulating tumour DNA; ER: oestrogen receptor; FES: fluoro-17β-estradiol; HER2: human epidermal growth factor receptor 2; PET/CT: positron emission tomography/computed tomography; TNBC: triple-negative breast cancer.

Taken together, ER-targeted PET, HER2-targeted PET, FDG PET/CT under immunotherapy, and ctDNA kinetics illustrate how whole-body and systemic biomarkers may help address clinically relevant grey zones in breast cancer management. However, their evidentiary maturity differs substantially. FES PET/CT has the strongest support as a functional ER-mapping tool in selected clinical scenarios, whereas HER2-targeted PET remains mainly a promising problem-solving approach for suspected receptor heterogeneity when tissue assessment is limited. FDG PET/CT can provide valuable whole-body metabolic information during immunotherapy, but atypical immune-related response patterns require cautious interpretation and breast cancer-specific validation of response criteria. Finally, combined FDG PET/CT and ctDNA monitoring offers a biologically compelling framework for response-adaptive care, but escalation and especially de-escalation strategies remain investigational. The near-term role of these tools is therefore most defensible in clearly defined clinical questions, biopsy prioritisation, multidisciplinary problem-solving, and prospective trials with predefined biomarker-guided actions (Figure 1).

## Abbreviations

[18F]FES: 16α-[18F]fluoro-17β-estradiol
18F-FDG: 18F-fluorodeoxyglucose
ctDNA: circulating tumour DNA
ER: oestrogen receptor
eTNBC: early triple-negative breast cancer
HER2: human epidermal growth factor receptor 2
ICIs: immune checkpoint inhibitors
iPERCIST: immune PERCIST
irAEs: immune-related adverse events
MRD: minimal residual disease
mTNBC: metastatic triple-negative breast cancer
MTV: metabolic tumour volume
pCR: pathological complete response
PET/CT: positron emission tomography/computed tomography
PR: progesterone receptor
RCB: residual cancer burden
SUL: standardized uptake value normalized to lean body mass
SUV: standardized uptake value
T-DM1: trastuzumab emtansine
TLG: total lesion glycolysis
TNBC: triple-negative breast cancer

## Supplementary materials

The supplementary figures for this article are available at: https://www.explorationpub.com/uploads/Article/file/1002398_sup_1.pdf.

## Declarations

### Copyright

© The Author(s) 2026.
