# Supplementary material for: Response-adaptive breast cancer care in the grey zones: integrating ER and HER2 targeted PET, FDG PET/CT under immunotherapy, and ctDNA kinetics
Source: Explor Target Antitumor Ther. 2026 Aug 16;7:1002398. doi: 10.37349/etat.2026.1002398 (PMC13485471; doi:10.37349/etat.2026.1002398)
Supplement: Supplementary file 1 [file 1002398_sup_1.pdf]

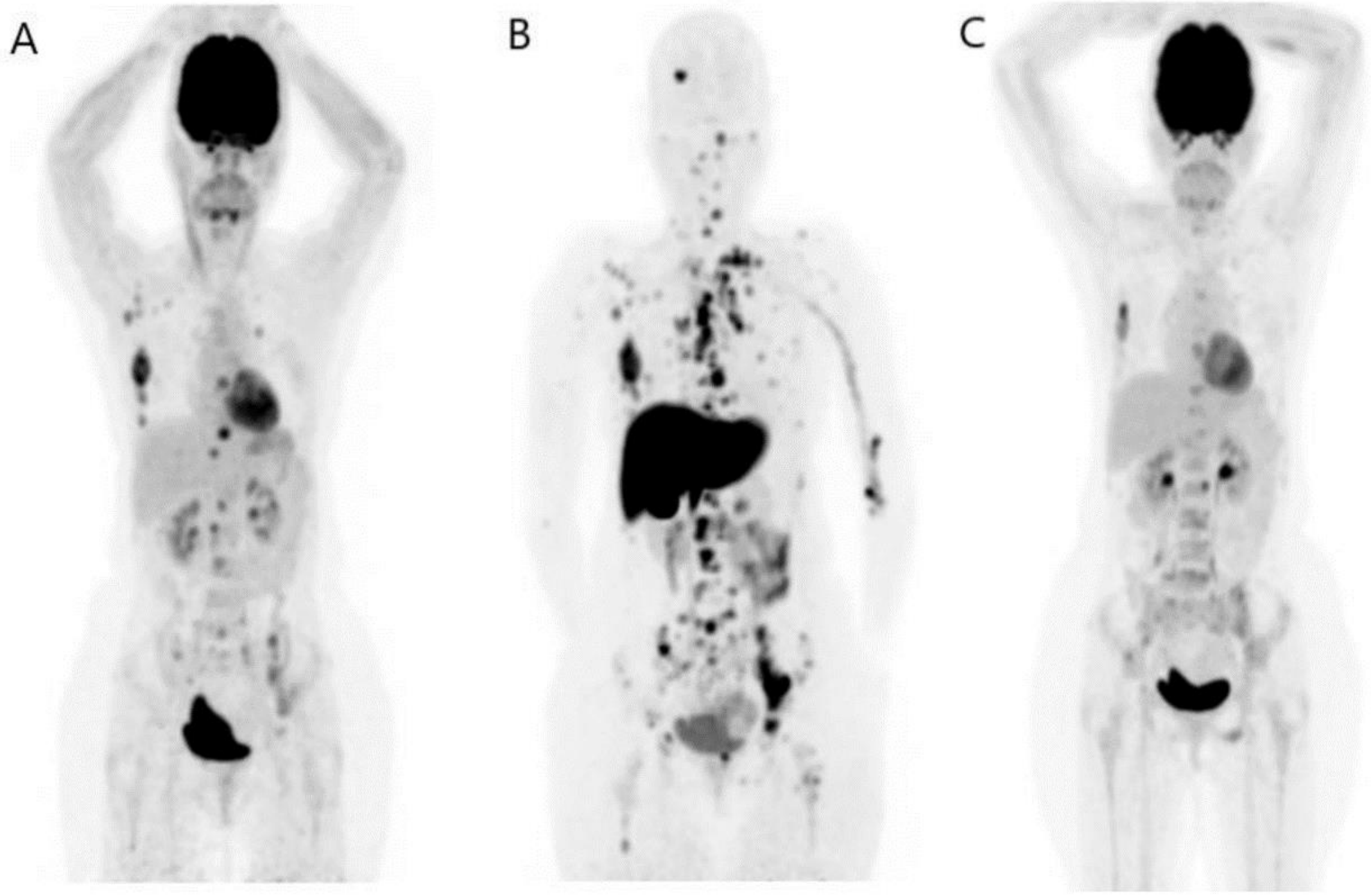

Figure S1. Example of FES PET/CT in treatment guidance. A 50-year-old woman was diagnosed with BR eight years ago and treated with surgery, chemotherapy, and radiotherapy. A recurrence was detected by FDG PET/CT (A for MIP image). As the tumor was initially RH+, treatment with fulvestrant was considered. A FES PET/CT was performed prior. Its MIP image (B) shows all lesions FDG+ were FES+ and it detected other FDG- lesions. At the evaluation of fulvestrant's treatment response 3 months later, the FDG PET/CT (C for MIP image) shows an important partial metabolic response. Reprinted from: Figure 6 in Zhang-Yin J. State of the Art in 2022 PET/CT in Breast Cancer: A Review. J Clin Med. 2023;12:968. Published 2023 Jan 27. CC BY. https://www.mdpi.com/2077-0383/12/3/968

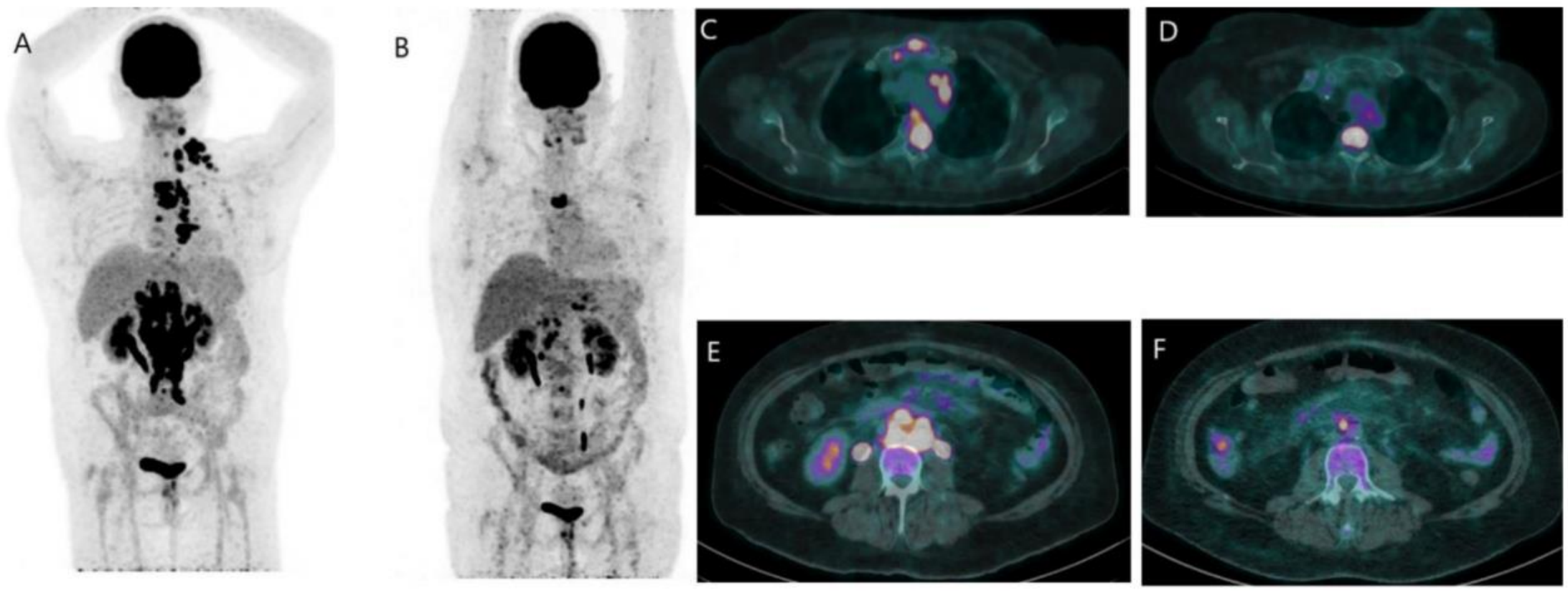

Figure S2. FDG PET/CT in evaluation of treatment response. A 47-year-old woman with metastatic HER2+ BR was treated by pertuzumab and was referred for evaluation of treatment response. The PET/CT MIP images (A,B) show a significant partial metabolic response at 3 months of interval. Images (C,D) (fusion image, trans-axial view) show the regression of mediastinal adenopathy and sternum lesion and also the persistence of a bone lesion of T4. Images (E,F) (fusion image, trans-axial view) show the important regression of retroperitoneal lymph node involvement. Reprinted from: Figure 3 in Zhang-Yin J. State of the Art in 2022 PET/CT in Breast Cancer: A Review. J Clin Med. 2023;12:968. Published 2023 Jan 27. CC BY. https://www.mdpi.com/2077-0383/12/3/968
